# P14 Preparing the future pharmacy workforce: competency-based undergraduate curricula for teaching, learning and assessment with a focus on antimicrobial stewardship

**DOI:** 10.1093/jacamr/dlae217.018

**Published:** 2025-01-23

**Authors:** Naomi Fleming, Gill Damant, Antonella Tonna, Sandra Martin, Ryan Hamilton, David Allison, Diane Ashiru-Oredope, Helen Root, Kate Shemilt, Kathryn Bullen, Kevin Frost, Mamoon Aldeyab, Molly Courtenay, Roger Harrison

**Affiliations:** NHS England, East of England, UK; NHS England, North West, UK; Robert Gordon University, Aberdeen, UK; University of Bradford, Bradford, UK; De Montfort University, Leicester, UK; University of Manchester, Manchester, UK; University College London, London, UK; UK Health Security Agency, London, UK; De Montfort University, Leicester, UK; Liverpool John Moores University, Liverpool, UK; University of Sunderland, Sunderland, UK; Pharmacy Department, Airedale NHS Foundation Trust, Keighley, UK; University of Huddersfield, Huddersfield, UK; Cardiff University, Cardiff, UK; University of Manchester, Manchester, UK

## Abstract

**Background:**

Competency-based teaching, learning and assessment underpins the requirements for initial training and education of pharmacists (IETPs) in the UK as set by the General Pharmaceutical Council (GPhC). This competency-based pedagogic approach is conducive when teaching antimicrobial resistance (AMR) and antimicrobial stewardship (AMS). It ensures that rather than only reciting and absorbing content, student pharmacists gain the necessary skills and behaviours to apply knowledge effectively in clinical practice. This is particularly relevant since foundation trainee pharmacists (in their fifth year after completing the pharmacy degree and before the pre-registration exam) need to show competency in independent prescribing at the point of registration from 2026. Therefore, education needs to evolve to incorporate prescribing skills including decision-making.

**Objectives:**

To develop an AMS competency framework specifically tailored for student pharmacists and relevant to the UK.

**Methods:**

A working group of academics and pharmacy practitioners from all four nations and with expertise in AMS was set up in September 2022 (NAPEG). Student pharmacists from a national organization (BPSA) and national pharmacy bodies including UKCPA, UKHSA, NHSE, NHS Wales, HIS, BSAC and RPS are members. Development of the consensus AMR and AMS competency framework for student pharmacists was coordinated by NHS England; relevant indicators from published frameworks (UK undergraduate medical students, and a UK-wide set of generic AMS competencies for undergraduate healthcare professional education) were included alongside new indicators developed by NAPEG. The results of a survey conducted across Schools of Pharmacy (SoP) aiming to determine the nature and extent of implementation of the latter generic competencies, also informed this project. To ensure consistency, the group worked with BSAC to update the Keep Antibiotics Working (KAW) website (which provides resources for pharmacy students and educators) and align to the framework domains. No ethics approval was required for this study since it did not involve any participants.

**Results:**

The final framework consists of six domains: Infection prevention and control, antimicrobials and antimicrobial resistance, antimicrobial prescribing and stewardship, vaccine uptake, person-centred care and interprofessional collaborative practice. Each domain includes a competency statement together with accompanying descriptors (74) clearly outlining the knowledge and application required by the newly qualified pharmacist. To support implementation of the framework, the individual competencies were mapped to the 2021 GPhC standards for the IETPs together with the RPS Prescribing Competency Framework. This indicative curriculum was published by NHSE Workforce, Training and Education Directorate. The group has also developed a set of suggested practice-based activities aligned with the framework domains to support pharmacists supervising or assessing students on placement.

**Conclusions:**

This project is timely to support development of future pharmacists as leaders in AMS and ensure a portable workforce, since pharmacists will be independent prescribers at the point of registration from 2026. A limitation of the development is the fact that not all SoP were involved in NAPEG; however, efforts are being made to communicate curriculum content as widely as possible. Though it is not mandatory to implement this curriculum, it provides a benchmark for embedding the competencies into undergraduate pharmacy curricula and allows identification of topics that may not be adequately covered. It also promotes consistency of approach across schools.

